# A visualization analysis of hotspots and frontiers of cardiovascular diseases with frailty

**DOI:** 10.3389/fpubh.2022.915037

**Published:** 2022-10-10

**Authors:** Xuping Bao, Loretta Yuet Foon Chung, Yujie Wen, Yifei Du, Qiyu Sun, Yi Wang

**Affiliations:** ^1^Evidence-Based Nursing Center, School of Nursing, Lanzhou University, Lanzhou, China; ^2^Department of Cardiovascular Medicine, Gansu Provincial Hospital, Lanzhou, China

**Keywords:** bibliometric analysis, visualization analysis, cardiovascular diseases, frailty, the elderly, exercise intervention, social frailty

## Abstract

Cardiovascular diseases (CVD) and frailty are common health problems among the elderly. This research aims to investigate the hotspots and frontiers of the field of CVD with frailty. Data of publications between 2000 and 2021 were collected from the Web of Science Core Collection (WoSCC) and CiteSpace was used for analyzing the hotspots and frontiers of cardiovascular diseases with frailty research from high-impact countries/regions, institutions, authors, cited references, cited journals, high-frequency keywords, and burst keywords. The results showed that the USA, England, and Canada were the leading countries/regions in research on CVD with frailty. Other countries/regions and regions lagged behind these developed countries/regions. There is a need to establish cooperation between developed countries/regions and developing countries/regions. Research hotspots focused on frailty in the elderly with CVD, exercise intervention, assessment for CVD patients with frailty, quality of life, and common diseases related to CVD with frailty. The frontier fields include care and intervention of CVD patients with frailty, social frailty, and validation of CVD with frailty.

## Introduction

Frailty is an age-related syndrome, which is characterized by multiple organ decline (1). When patients are exposed to frailty, they will be more sensitive to stress and their ability to cope with the stress may also decrease (1). As a result, it will lead to adverse events in hospitals, such as falls, disability, and even death. Not only hospitalized patients may suffer from frailty but also community-dwelling people. Community-dwelling people with frailty have a high risk to suffer from negative events, such as disability, hospitalization, death, and so on (2). A study in Spain which consisted of 855 individuals found that the prevalence of frailty among community-dwelling people was 26.2% (3), and increased frailty prevalence was also related to worse comorbidity status (3). Hence, not being admitted to the hospital should not become a reason for reducing public awareness of frailty. Frailty is a common problem in aged people (4). With the increase in aged population, there will be more frail people (5).

Cardiovascular disease (CVD) is the leading cause of disability and death among the elderly. Meanwhile, it also leads to rising healthcare costs. According to the data from the Global Burden of Disease, the number of patients with CVD doubled from 271 million in 1990 to 523 million in 2019 globally, and the case of death increased from 12.1 million in 1990 to 18.6 million in 2019 (6). The aging population and frailty are the major reasons for the increase in CVD. Clearly, the main research objective population of CVD with frailty is the elderly.

Nearly one in two adults with heart failure suffers from frailty (7). Chung et al. found that patients with advanced heart failure and handgrip strength < 25% of body weight had increased postoperative complications and risk of death after ventricular assist device implantation (8). In frailty phenotype (FP), the decreased grip strength is one of the factors (9). Besides, the CVD patients with frailty showed functional dependency and a poor quality of life compared to the non-frail in a 1-year follow-up study (10). In addition, higher Framingham CVD risk scores were associated with an increased risk of incident frailty (11). Therefore, frailty is associated with poor health in patients with CVD. Hence, a lot of researchers also identified frailty as a risk factor for CVD. Identifying the frailty of CVD patients helps to give the most suitable care to the different types of patients. Research has shown that frailty has different statuses in different diseases, and specific critical value of frailty status in one group of patients may not be applicable in another group, so the author suggested that researchers should define the optimal toolset to measure frailty (12). Therefore, researching the frail status of patients with CVD is a high priority.

Frailty is not a disease; instead, it is similar to disability and complications. CVD with frailty is not only a medical problem but also a public health challenge because both CVD and frailty exist in the elderly population in society widely. For hospitalized CVD patients, discerning early frailty can prevent adverse events, and taking the status of frailty into medical and nursing plans also can lead to a better treatment outcome. For community-dwelling people with chronic CVD, screening frail individuals and developing interventions related to lifestyle by community doctors and nurses are effective measures to decrease admission and readmission rates, which can reduce medical costs. A retrospective study result found that continuous physical interventions may improve the functional status of patients with CVD from admission to 6 months after discharge, which was beneficial for reversing frailty in the patients (13). In addition, cardiac rehabilitation improves physical function and reduces rehospitalizations in patients with CVD after discharge (14, 15). Consequently, CVD patients with frailty should get more attention than common CVD patients. First, it is urgent to evaluate the impact of scholarly research in this domain, as well as the impact of countries/regions, authors, journals, and institutions. Moreover, exploring the current topics and identifying the problems that can inspire later researchers to have further research to promote public health.

In the existing reviews, some authors focus on the relationship between frailty and CVD. For example, Uchikado et al. expounded on the influences of frailty in common CVD, such as coronary artery disease, heart failure, aortic stenosis, and atrial fibrillation (12). Marinus et al. found that frailty is the most common problem among patients with heart failure or aortic valve disease in a systematic review (16). Most authors discuss CVD with frailty in their traditional literature review. In recent years, some researchers probed the prevalence and effect of frailty in CVD patients by systematic review; however, none of them has taken the visualization analysis. In addition, although a literature review is a basic measure to understand the development in a specific research area, the authors in the reviews may have certain preferences on some aspects they were previously interested in. As a result, they may ignore some topics and cannot present a complete view sometimes. To obtain a further survey of the development of the CVD with frailty roundly and intuitively and to fill a gap in visualization analysis, a visualization knowledge map was adopted in this study.

CiteSpace is a widely used information visualization software established by professor Chen (17), and it can intuitively show the hotspots, frontiers, and growth of literature in a specified area of academic research. In CiteSpace, research hotspots in this field can be presented through images intuitively, as well as the clear connections among nodes. Thus, CiteSpace was chosen as the tool in this research.

This research aims to discuss the hotspots and frontiers in the CVD with frailty. High-impact countries/regions, institutions, authors, cited authors, references, journals, high-frequency keywords, and burst keywords were shown by CiteSpace to achieve the goal of the research. The analysis of this topic will contribute to new research perspectives in future.

## Methods

### Search strategy

The data for this study came from the Web of Science Core Collection (WoSCC). WoSCC was chosen for this study because it is a large comprehensive multidisciplinary database that covers 18,000 most influential journals around the world. WoSCC assigns document type labels to the publications more accurately than alternatives such as Scopus (18). The search strategy was as follows: [TS = (frailty OR frail) AND TS = (“cardiovascular disease”)].

### Inclusion criteria

The type of records was limited to the article and the language was English. The articles published on the WoSCC from 2000 to April 2021 were obtained. Initially, 719 articles were collected from WoSCC. Then, two independent researchers read the titles and abstracts and removed the records that were not related to CVD with frailty. After reading the titles and abstracts, 133 eligible records were brought into this study.

### Data and analysis

**Figure 1:**
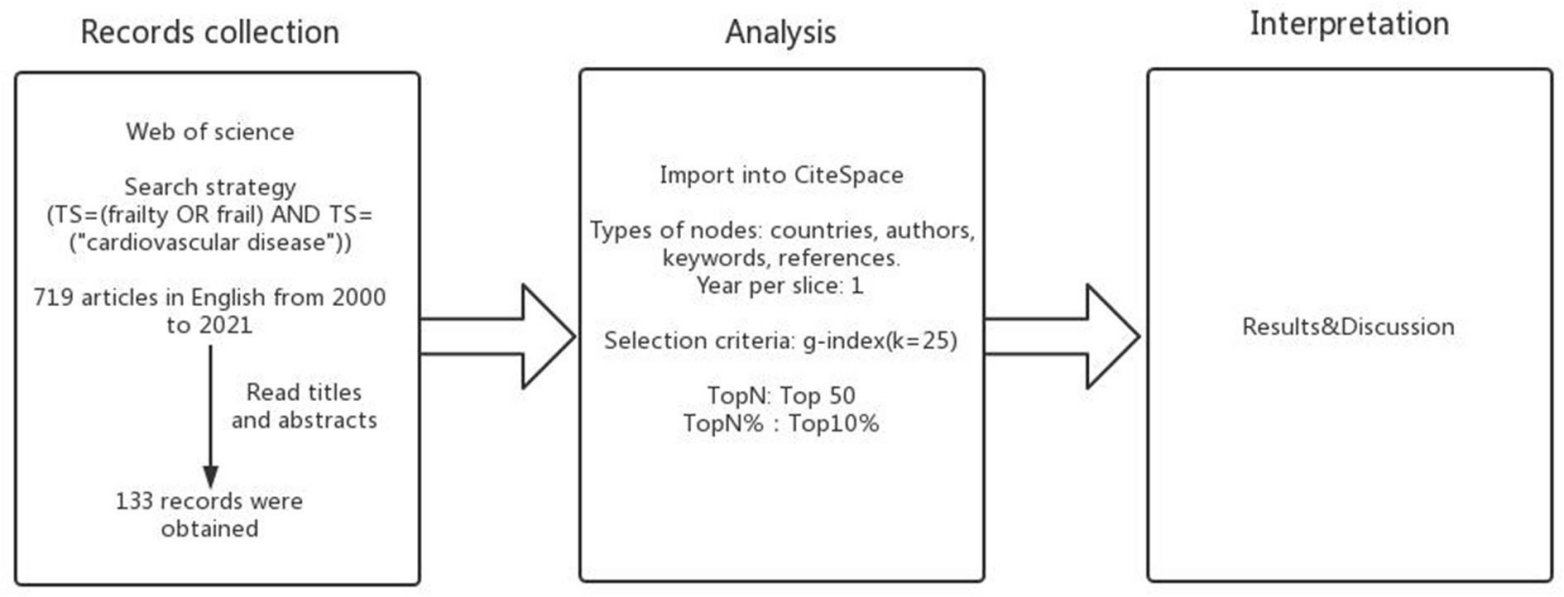
Overview of the study protocol.

The 133 records were imported into CiteSpace for further analysis. The year per slice was 1 year. The types of nodes were author, institution, country/region, keyword, reference, cited author, and cited journal. The top 50 levels of most citations from each year were selected during the process of analysis of countries/regions, institutions, authors, and keywords, and the g-index (k = 25) was selected to analyze the cited references, cited journals, and cited authors. Pathfinder, pruning sliced networks, and pruning the merged network were selected in the module of pruning. These parameter settings had no deviations from default settings. Co-occurrence and cluster views were chosen to present the images. Then, CiteSpace was used to analyze the hotspots and frontiers from high-impact countries/regions, institutions, authors, cited references, cited journals, high-frequency keywords, and burst terms. In the tables, influences of countries/regions, institutions, and authors are presented from frequency and centrality viewpoints. In the figures, the sizes and colors of nodes are key points. Figure 1 shows this process as follows.

## Results

Included in this study were 133 publications from 2000 to 2021.

### Analysis of countries/regions, institutions, and authors

**Figure 2:**
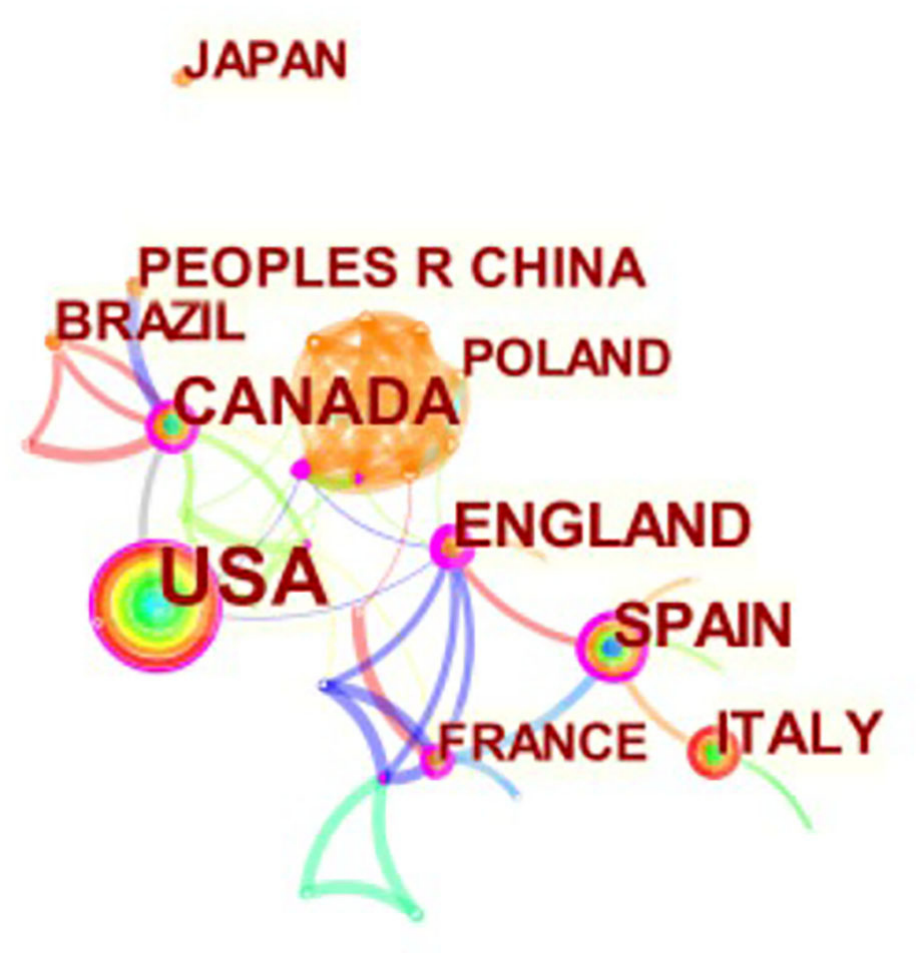
Map of countries related to CVD with frailty from 2000 to 2021.

**Figure 3:**
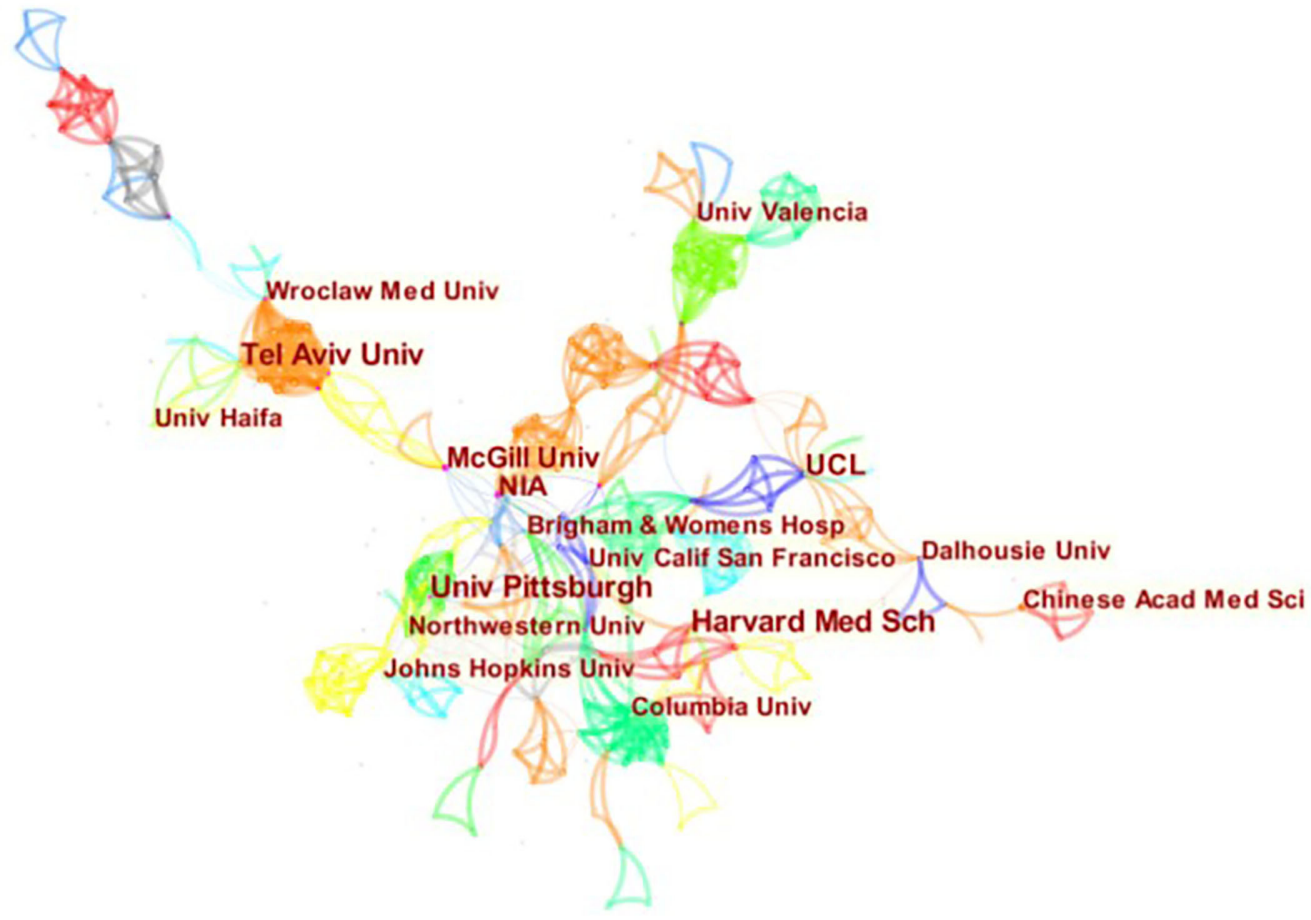
Map of institutions related to CVD with frailty from 2000 to 2021.

**Figure 4:**
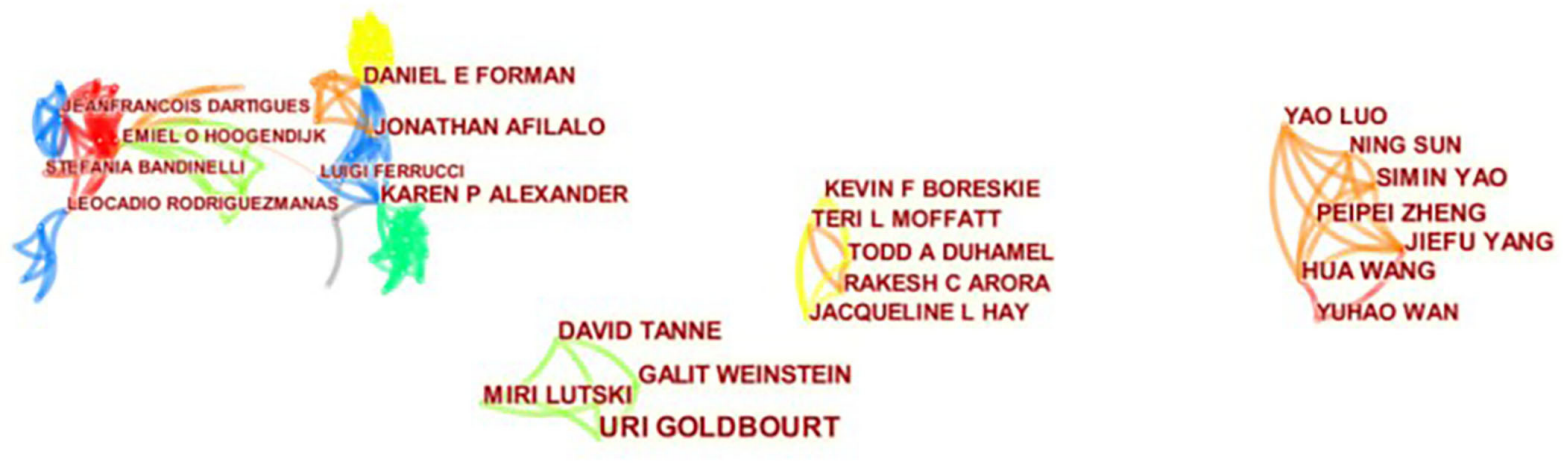
Map of authors related to CVD with frailty from 2000 to 2021.

Table 1, Figures 2–4 show the top 10 countries/regions, institutions, and authors according to the number of publications on CVD with frailty. Figure 2 consists of 39 nodes and 131 collaboration links. The USA has the largest number of articles (*n* = 41), the second is Canada (*n* = 17), and the third is England (*n* = 14). Although the USA is way ahead of the other countries/regions in numbers, its centrality is not the highest. High centrality means that the node plays an important role in the specific field. Based on centrality, the top three countries/regions are England, Spain, and Canada, which means that these countries/regions are the most influential in the research domain of CVD with frailty. In addition, although the number of articles in France is < 8 in other countries/regions, its centrality is at a high level (0.25), which indicates that the quality of the articles is high. The purple circle of nodes in Figure 2 is the key marker, and it means that the centrality of the country/region was high, which indicates that these countries/regions play an important role in this field. These countries/regions are the USA, England, Spain, Canada, and France. Figure 3 consists of 359 nodes and 1,081 collaboration links. The top three institutions based on frequency are Harvard Medical School, Tel Aviv University, and University of Pittsburgh, and two of them are in the USA. The top three institutions based on centrality are NIA (National Institute on Aging), McGill University, and University of Pittsburgh. Both the number and the centrality of publications of the University of Pittsburgh are in the top three in the world. In Figure 3, the nodes of NIA and McGill University are surrounded by a purple circle, indicating that these two institutions have greatly contributed to CVD with frailty research. Figure 4 consists of 903 nodes and 2,826 collaboration links. Given that the nodes of authors are dispersive in the map, a part of the concentrated distribution of the whole map is shown in this research. Nine prolific authors come from Israel and China.

**Table 1:** Top ten countries/regions, institutions and authors according to the frequency of articles related to CVD with frailty.

**Countries/regions**	***n***	**Centrality**	**Institution**	***n***	**Centrality**	**Author**	***n***	**Centrality**
The USA	41	0.13	Harvard Medical School, The USA	6	0	Uri Goldbourt, Israel	5	0
Canada	17	0.28	Tel Aviv University, Israel	6	0.04	Galit Weinstein, Israel	4	0
England	14	0.56	University of Pittsburgh, The USA	6	0.11	Peipei Zhang, China	4	0
Spain	14	0.34	University College London, England	5	0.09	Jiefu Yang, China	4	0
Italy	11	0.09	McGill University, Canada	5	0.27	Hua Wang, China	4	0
China	10	0	NIA (National Institute on Aging), The USA	5	0.31	Nicola Veronese, Italy	4	0
Brazil	10	0	University of Haifa, Israel	4	0	David Tanne, Israel	4	0
Japan	9	0	University of California, San Francisco, The USA	4	0.02	Miri Lutski, Israel	4	0
France	8	0.25	Chinese Medical Association, China	4	0.02	Simin Yao, China	4	0
Poland	8	0	Columbia University, The USA	4	0.03	Daniel E Forman, The USA	3	0

### Analysis of cited authors, references, and journals

Co-citation analysis is the unique advantage of CiteSpace which not only explores the influential authors, references, or journals but also assists the hotspots in a specific study area. The co-citation explores in this research used a g-index (k = 25) for analysis. Table 2 shows the cited authors, references, and journals that are related to CVD with frailty.

**Table 2:** Cited authors, references and journals related to CVD with frailty.

**Cited authors**	**Count**	**Centrality**	**Cited references**	**Count**	**Centrality**	**Cited journals**	**Count**	**Centrality**
Fried LP, Columbia University, The USA	104	0.09	Afilalo et al. (20)	22	0.25	The American Journal of Medicine	30	0.23
Afilalo J, McGill University, Canada	73	0.13	Clegg et al. (21)	20	0.06	Circulation-Cardiovascular Quality and Outcomes	29	0.03
Newman AB, The University of Pittsburgh, The USA	54	0.07	Singh et al. (22)	15	0.15	International Journal of Cardiology	28	0.03
Clegg A, University Leeds, England	48	0.12	Sergi et al. (23)	15	0.07	Clinics in Geriatric Medicine	28	0.09
Rockwood K, Dalhousie University, Canada; Xuanwu Hospital, China	48	0.15	Veronese et al. (24)	10	0.08	BMJ-British Medical Journal	26	0.1
Singh M	30	0.07	Afilalo et al. (25)	10	0.08	Clinical Interventions in Aging	23	0.01
Walston J, The John Hopkins Medical Institutions, The USA	23	0.06	Afilalo (26)	9	0.1	European Journal of Clinical Investigation	23	0.02
Cacciatore F, Salvatore Maugeri Foundation, Institute of Care and Scientific Research, Italy	22	0.15	Ekerstad et al. (27)	9	0.17	Aging Clinical and Experimental Research	22	0.7
Purser JL, Center for the Study of Aging and Human Development, The USA	19	0.02	Soysal et al. (28)	8	0.01	BMC Medicine	20	0.01
Lee DH, Canadian Cardiovascular Outcomes Research Team, Canada	19	0.06	Singh et al. (29)	8	0.03	Journal of Clinical Epidemiology	20	0.01
–	–	–	–	–	–	BMJ Open	19	0.01
–	–	–	–	–	–	Geriatrics & Gerontology International	19	0.05

**Figure 5:**
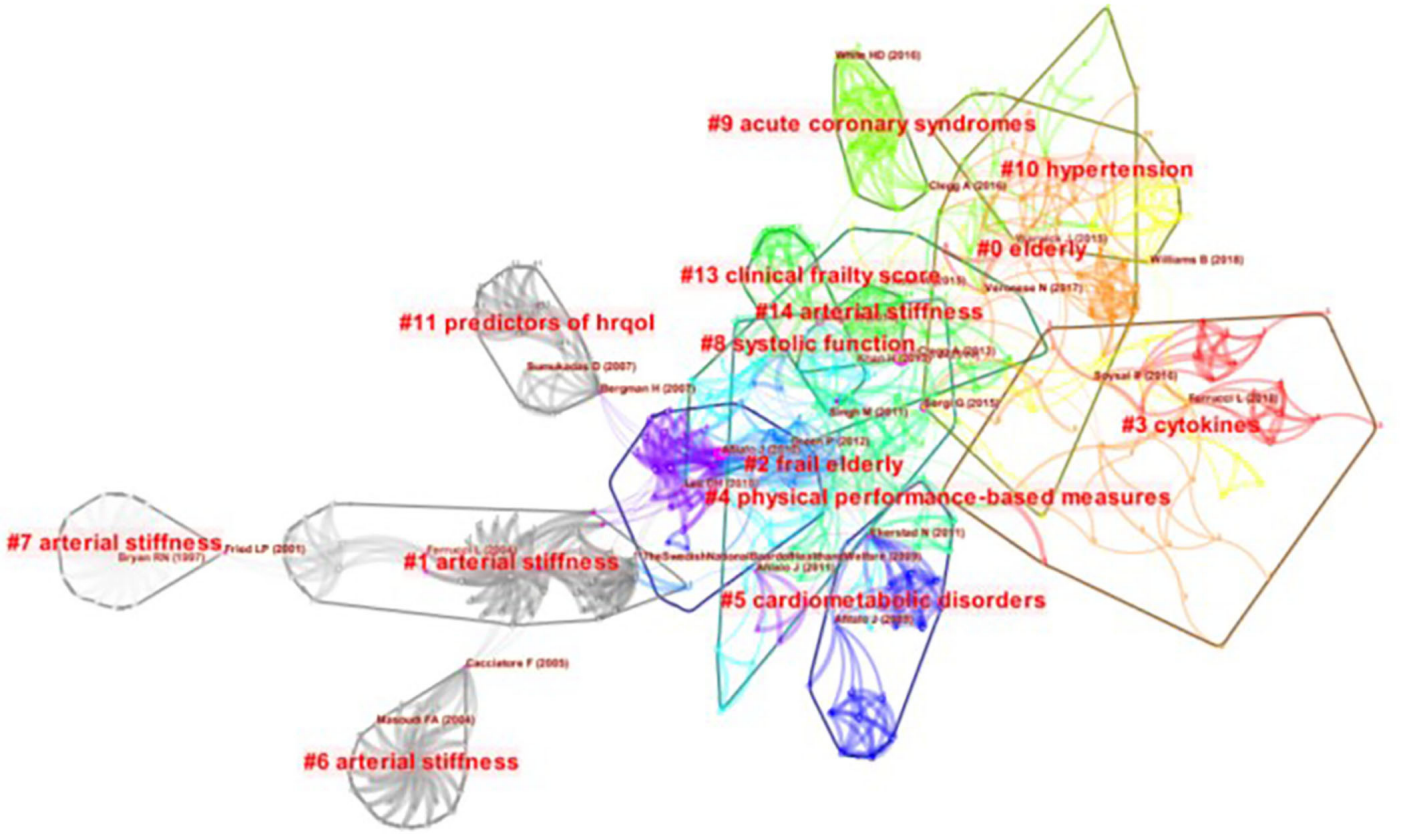
Clusters of cited references related to CVD with frailty from 2000 to 2021.

Professor Fried LP, who comes from an authoritative institution for CVD with frailty, is the most cited author related to CVD with frailty (*n* = 104). Professor Afilalo J of McGill University is the second most cited author related to frailty with CVD and three of his articles were the top 10 cited references related to CVD with frailty. In addition, Rockwood K from Dalhousie University and Cacciatore F from the Institute of Care and Scientific Research of Italy have the highest centrality. The cited references reflect the long-term hotspots in a specific field (19), hence, clusters map of cited references related to CVD with frailty is presented in Figure 5, where the software works out 33 clusters. After limiting the clusters automatically by the software, there are 14 significant clusters in total. The result of cluster map is convincing because the map shows that Q = 0.85 > 0.3, S = 0.95 > 0.5 (17). The top five clusters are #0 elderly, #1 arterial stiffness, #2 frailty elderly, #3 cytokines, and #4 physical performance-based measures. The top 10 cited journals are medical journals, with geriatrics and cardiovascular journals dominating. These journals can be called the “core journals” in this field. The publishing countries/regions are all developed countries/regions, and the UK has the most cited journals among the top 10 journals related to CVD with frailty. The American Journal of Medicine has the highest frequency and centrality simultaneously.

### Analysis of keywords

**Figure 6:**
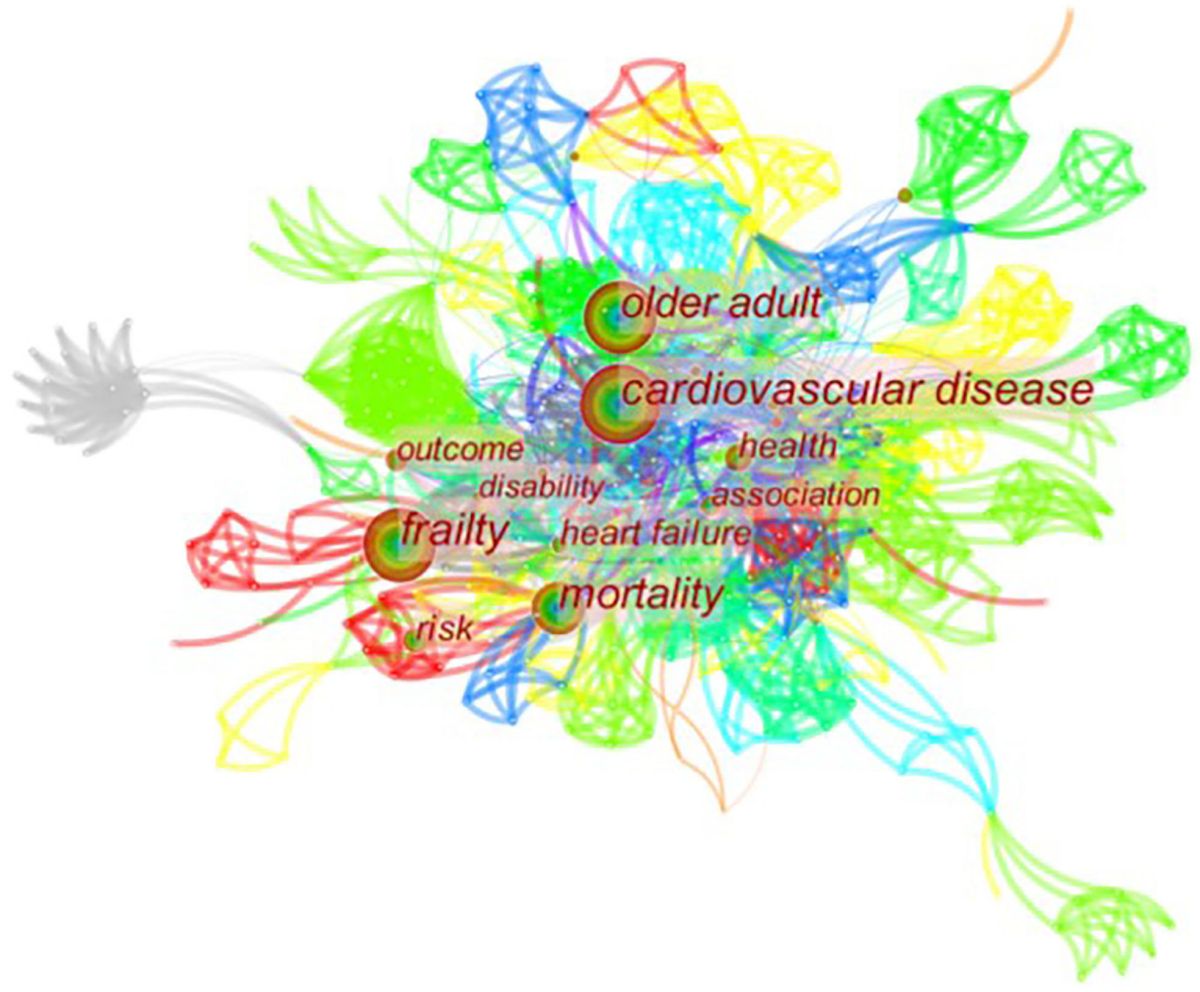
Map of keywords related to CVD with frailty from 2000 to 2021.

**Figure 7:**
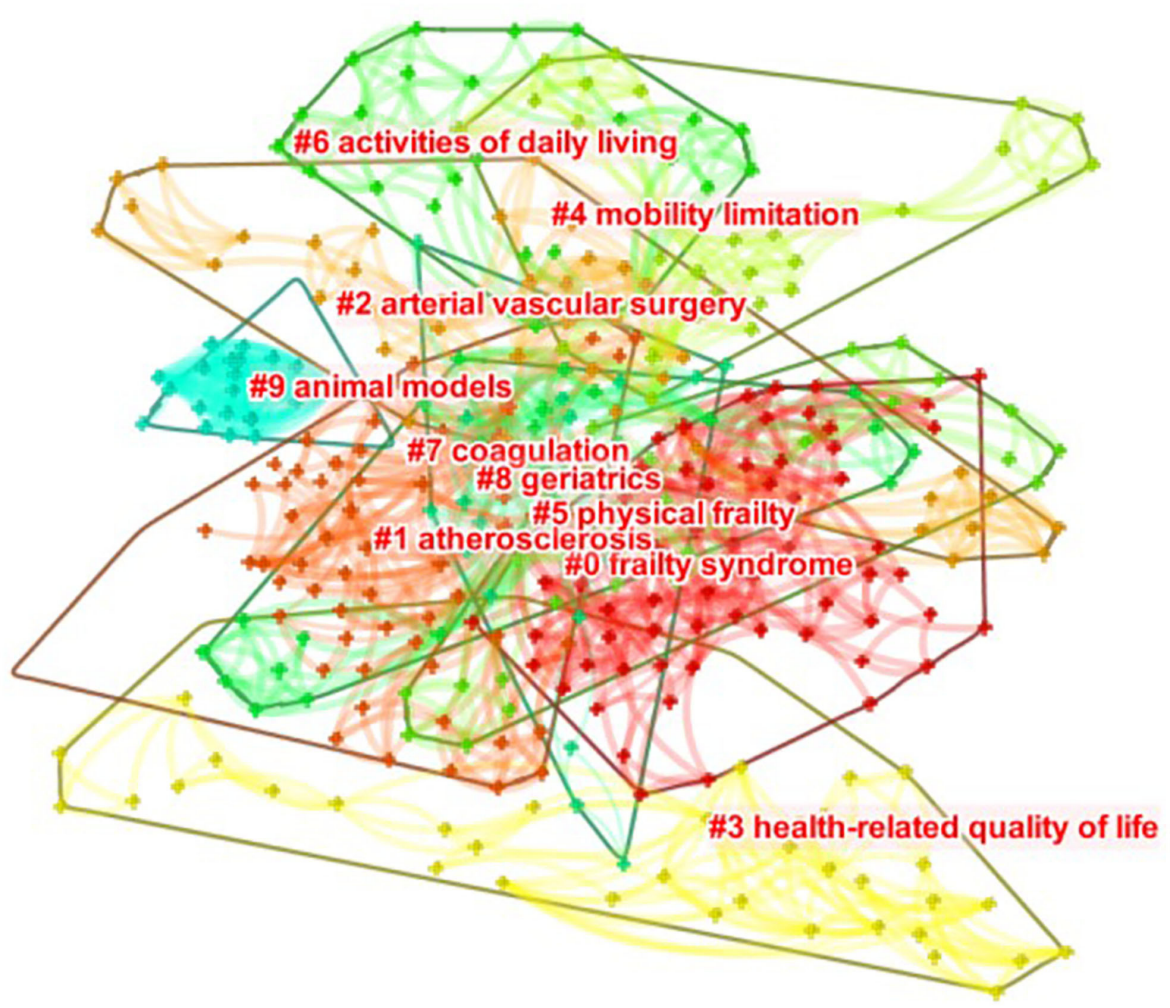
Clusters of keywords related to CVD with frailty from 2000 to 2021.

The researchers merged the keywords that share the same meaning and graphed the visual figure of the keywords related to CVD with frailty (Figure 6). Figure 6 consists of 480 nodes and 2,506 collaboration links. According to frequency, the top 10 high-frequency keywords in this field were sorted in Table 3. The top five high-frequency keywords are “frailty,” “cardiovascular disease,” “older adult,” “mortality,” and “health.” The top three high-centrality keywords are “cardiovascular disease,” “mortality,” and “disability.” The nodes are bigger than the others in Figure 6, and the frequency of keywords is higher than the others, which means they are the hotspots in this field. Then, a clusters map of keywords related to CVD with frailty was graphed in Figure 7, and 23 clusters were presented in total. The result of cluster map is convincing because the map shows that Q = 0.69 > 0.3, S = 0.90 > 0.5 (17). The top 10 clusters related to size were selected to make the cluster map clear in Figure 7. The top five clusters are #0 frailty syndrome, #1 atherosclerosis, #2 arterial vascular surgery, #3 health-related quality of life, and #4 mobility limitation.

**Table 3:** Top high frequency keywords related to CVD with frailty.

**Keywords**	**Count**	**Centrality**	**Year**
Frailty	85	0.01	2008
Cardiovascular disease	80	0.13	2008
Older adult	66	0	2009
Mortality	57	0.07	2001
Health	28	0.05	2001
Heart failure	27	0.01	2008
Risk	26	0	2014
Association	24	0.04	2013
Outcome	23	0.04	2012
Disability	20	0.06	2005

### Analysis of keywords of bursts

**Figure 8:**
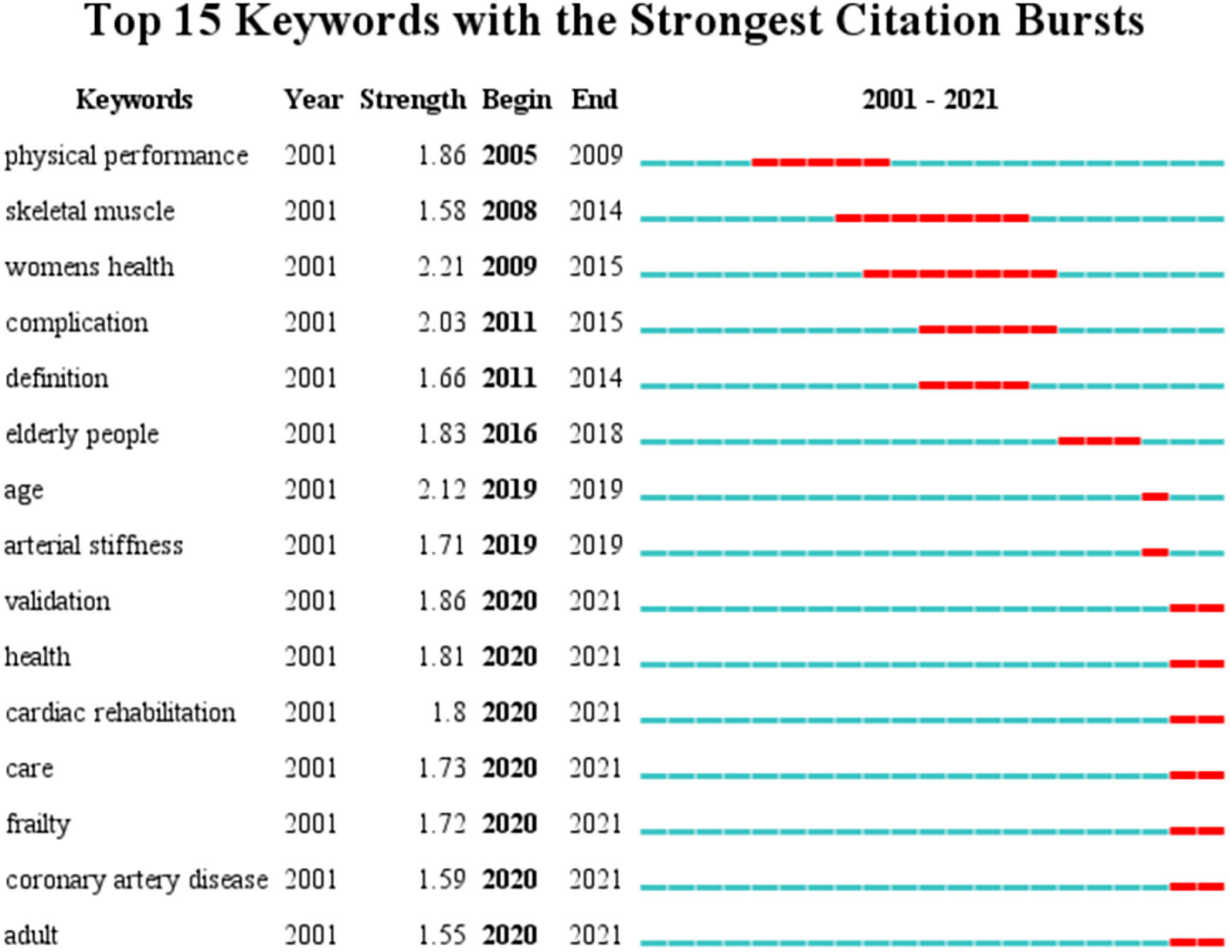
Top 15 keywords of bursts related to CVD with frailty from 2000 to 2021.

Fifteen keywords of bursts are shown in Figure 8, and the minimum duration is 1 year and γ = 0.5. The top three strongest bursts were “women health (2.21),” “age (2.12),” and “complication (2.03).” Sigma (∑) measures the combined strength of the structural and temporal properties of a node, its betweenness centrality, and citation burst. Burst strength is computed through centrality and ∑, with higher values indicating higher influential potential. In this study, ∑ is 1.00. There are more bursts in 2020 than in other years. There are nine keywords of bursts in nearly 2 years, and seven keywords have high strength in 2020. They are “validation,” “health,” “cardiac rehabilitation,” “care,” “frailty,” “coronary artery disease,” and “adult.”

## Discussion

In this research area, the USA, England, and Canada are the leading countries/regions. Although some developing countries/regions such as Israel and China have been paying great attention to CVD with frailty, they continue to show deficiencies in the quality of studies. The study highlighted the hotspots and frontiers and presented several clusters in the research area of CVD with frailty. Older adults with CVD, exercise intervention, assessment for CVD patients with frailty, quality of life, and common diseases related to CVD with frailty are concerns among authors in this research domain. In recent years, researchers turn their attention to interventions for CVD patients with frailty. Moreover, care and intervention of CVD patients with frailty, social frailty, and validation of CVD with frailty will be the trending research topics in future.

### Development of top 10 countries/regions

England and Canada lead the world in the domain of CVD with frailty by synthesizing the number of articles and centrality. The top five countries/regions by the number of publications in CVD with frailty are developed countries/regions. This may be due to a faster increase in the aging population compared to other developing countries/regions. Although population aging is a global phenomenon, the situation differs among countries/regions (30). There are two Asian counties in the top 10 prolific countries/regions in CVD with frailty. It is mostly developing countries/regions in Asia; indeed, the medical resources and environments in developing countries/regions are not at a high level. Sometimes, doctors and nurses may ignore the accompanying symptoms which are not obvious such as unintentional weight loss, weakness, and so on, because they focus on the disease therapy more. Generally, the frailty of CVD patients is ignored in Asian countries/regions. Besides, Figure 2 shows that cooperative relationship in this field was limited to only a few countries/regions, thus cooperation between countries/regions should be strengthened in future.

### Contribution of institutions

According to Figure 3, close cooperation exists between various institutions. Universities made up the majority of the top 10 institutions, and most of these institutions are from developed countries/regions—half of them belonging to the USA, which is in accord with the top 10 countries/regions (Table 1). NIA and McGill University are also influential in CVD with frailty. The analysis of institutions provides the basis for talent introduction and further study.

### Contribution of individuals

As shown in Table 2 and Figure 4, most of the top 10 authors by the number of articles related to CVD with frailty are from Israel and China, which indicates that some Asian countries/regions pay attention to CVD with frailty. The number of prolific authors' publications is similar among the top 10 authors by the number of articles, and several of these authors come from the same institutions. The authors prefer to first collaborate with a prolific author and form a co-author cluster afterward. Besides, in terms of country/region and institution, it is the European and American countries/regions that had the advantage in the numbers of articles. That is to say that Asian countries/regions were in their preliminary stage in this field and do not have influential and leading experts or teams. Asian countries/regions should establish multicentric cooperation with developed countries/regions to guide their research of CVD with frailty.

### Co-citation analysis

Fried LP from Columbia University is the most cited author (Table 2), and he put forward FP based on data from the cardiovascular health study in 2001 that is used widely. FP is a diagnostic criteria of frailty including five items: unintentional weight loss, self-reported exhaustion, weakness (grip strength), slow walking speed, and low physical activity (9). People who have the clinical syndrome in three or more of the mentioned criteria are diagnosed with frailty. FP is an easy and convenient clinical assessment tool for frailty; however, it only has five items which is inadequate for assessments of potential frail patients. Meanwhile, the simplicity and feasibility of the assessment criteria contribute to its widespread use in the primary clinical screening for frailty. Professor Rockwood K from Dalhousie University has the highest centrality among the top 10 most cited authors (Table 2). In 2001, he developed a method for appraising health status in the elderly: frailty index (FI), consisting of 92 items that include symptoms, abnormal laboratory values, disease classifications, and disabilities (31). FI is more comprehensive compared with FP. It is because it includes more items that lead to a more all-sided prediction for frail adverse outcomes, meanwhile, it limits the use of FI in clinic practice. In general, both authors contributed to frailty assessment. Besides, professor Rockwood K established academic cooperation with Xuanwu Hospital of China to promote in-depth cooperation between China and Canada. In addition, professor Afilalo J of McGill University contributes to the basic theory review about CVD with frailty in all aspects.

What researchers focus on can be presented by cited references, and this content will be discussed in detail in the following section.

There are three geriatric journals among the top 10 cited journals (Table 2), which indicates that researchers attached great importance to elderly CVD patients with frailty. In other words, it is because CVD with frailty is common among the elderly that researchers focus on geriatric journals. One of the most influential journals, the American Journal of Medicine, is the official journal of the Alliance for Academic Internal Medicine which is a group comprising internal medicine department chairs at more than 125 medical schools across the USA. Its impact factor is 4.529 in June 2020. Frailty should be viewed as a reason for nursing in a more patient-centered fashion (21). As shown in Table 2, there are no nursing journals among the top 10 cited journals. However, frailty should be included during the whole process of nursing; optimal nursing pathways can relieve frailty. In the future, what nurses can do for CVD patients with frailty should be taken into consideration, and then conduct research from the nursing point of view.

### Hotspots in the CVD with frailty

The hotspots in a specific field are determined by co-occurrence figures, high-frequency keywords, and high-centrality keywords. To focus on the major topics, the contents in Table 3 and Figures 5–7 were summarized into five aspects as follows.

#### Frailty in the elder adults with CVD

The relevant keywords are “elderly” (Figure 5), “frail elderly” (Figure 5), “older adult” (Table 3), and “geriatrics” (Figure 7). Elder adults have been the focus of frailty and CVD. Nearly one-third of elderly inpatients have frailty, which is related to the length of hospital stay. The longer time of hospital stay, the higher possibility for the patient to have frailty (32). Most geriatric cardiovascular diseases are chronic, and most of the elderly with CVD have to remain hospitalized for a long time. It follows that the elderly with CVD are more probable to have frail than other populations. It is necessary to attach more importance to the elder adults with CVD, and elderly CVD patients with frailty will be a long-term hot research topic in future.

#### Exercise intervention for CVD patients with frailty

The relevant keywords are “activities of daily living” (Figure 7) and “mobility limitation” (Figure 7). Cesari et al. found that regular physical activity may reduce frailty, especially in individuals at higher risk of disability (33). Kehler et al. discovered that insufficient physical activity and prolonged sedentary time were detrimental despite CVD status (34). In other words, physical activities are beneficial to improve the remission situation of frailty and CVD. It will be a tendency to make a specific physical activity plan for CVD patients with frailty. Suitable physical activities are effective ways to improve the physical status of CVD patients with frailty, but specific details of the physical activities, such as time, frequency, and types are unknown. Therefore, it is needed to consider frailty and CVD simultaneously, making appropriate activity plans for this type of patients in intractable research.

#### Assessment for CVD patients with frailty

The relevant keywords are “physical performance-based measures” (Figure 5), “clinical frailty score” (Figure 5), and “frailty syndrome” (Figure 7). There are many assessment tools for CVD patients with frailty in the clinical practice, and the common tools are FP, FI, Clinic Frail Score (CFS), Tilburg Frailty Indicator (TFI), FRAIL, and so on. FP is one of the most widely used tools, and it works efficiently in primary clinical screening for frailty. FI is another common tool for frailty assessment (31); however, time investment to finish 92 items of FI may limit its use in busy clinical practice. Based on the FP and FI, FRAIL (35) gives a score according to specific symptoms, then assesses patients' frailty situation according to scores. CFS (36) determines patients' frail status according to the subjective judgment of evaluators. TFI (37) is a self-reported tool for screening the frail population in the community. It is a long-term therapy process for CVD patients with frailty, therefore, working out a settled assessment measure that can be used in daily clinical assessment is in greater need. Besides, in FP, only the options of “yes” and “no” are available for patients, and the condition of CVD patients with frailty is always complicated, so screening frailty according to a score will be a better choice for CVD patients. To recognize frailty accurately, both subjective and objective information should be obtained during the assessment process. However, spending too much time may become a limitation for clinic practice. Researchers use different instruments in studies about CVD patients with frailty at present, but it is convenient to evaluate the frailty status of CVD patients if a tool that can be called the “golden standard” will be developed in this field. Developing a more suitable instrument and selecting an applicative tool to discern frailty for different CVD patients have been brought into focus in this domain.

#### Quality of life of CVD patients with frailty

The relevant keywords are “predictors of HRQOL” (Figure 5), “health” (Table 3), and “health-related quality of life” (Figure 7). A cross-sectional, observational study suggested that frailty was independently associated with decreased quality of life (*p* < 0.0001) in the population with CVD (38). Good quality of life is the ultimate goal of medical and nursing care, but both CVD and frailty are two of the risk factors for decreased quality of life. To achieve the nursing care goal, it is necessary to be concern about the frailty of CVD patients. The assessment tools of quality of life are unambiguous; therefore, determining whether the frailty situation is improved by evaluating the quality of life is available.

#### Diseases related to CVD with frailty

The relevant keywords are “acute coronary syndromes” (Figure 5), “hypertension” (Figure 5), “cardiometabolic disorders” (Figure 5), “heart failure” (Table 3), and “atherosclerosis” (Figure 7). Many research found that frailty was common in older heart failure inpatients and it should be considered as a prediction factor to help identify individuals with an increased risk of mortality or readmission (39, 40). Some CVD may transform into heart failure eventually; hence, patients with heart failure are common among patients with CVD. Therefore, heart failure is a research hotspot in this field. The most cited author, Afilalo et al. expounded on the relationship between common CVD and frailty (20). During the whole process of therapy, although frailty is not a kind of disease, it is a key factor influencing the clinical outcome, which can lead to adverse events. Frailty should not be ignored no matter which kind of CVD patients have.

### Frontiers in the CVD with frailty

As shown in Figure 7, 15 burst keywords were considered as indicators of research frontiers. In terms of the time evolution of these keywords, the transformation of research frontiers can be summarized as follows: performance and definition of frailty (2005–2015), such as keywords “physical performance,” “skeletal muscle,” “complication” and “definition,” care and intervention of CVD patients with frailty (2020), and keywords such as “health,” “cardiac rehabilitation,” and “care.”

In addition, Quach et al. put forward the importance of social frailty in older adults (41); moreover, social frailty can be defined as a continuum of being at risk of losing or having lost social and general resources, activities, or abilities that are important for fulfilling one or more basic social needs during daily life in Bunt S's paper (42). Although there are social factors during the process of assessment of frailty, it is not enough to recognize social frailty and clinical tools for identifying social frailty remain unclear. Most of the time, geriatric cardiovascular diseases need a long time of therapy; thus, it is easy to have social frailty problems for older adults. However, the incorporation of another assessment into an already busy clinical practice should also be considered (41). As is shown in Figure 7, physical frailty is a hot topic in this field; however, with the gradual attention to humanistic care of CVD patients with frailty, social frailty will be a concern.

In other words, after synthesizing information in Figure 7, the CVD with frailty research frontiers are summarized as follows: care and intervention of CVD patients with frailty, social frailty, and validation of CVD with frailty.

### Limitations

First, since CiteSpace cannot process data from multiple databases, the analysis was limited to articles in the WoSCC database, which may have resulted in a publication bias. Second, to better present the results of the analysis and to ensure the quality of the included literature, only articles published in English were included. This may have led to a bias. In future, a more detailed visualization analysis should be available, for example, an analysis of articles in other languages such as Chinese, Spanish, German, and so on.

## Conclusion

This study aims to conduct a scientometrics analysis of CVD with frailty. It mainly included the contributory countries/regions, institutions, and authors, as well as the most cited authors, references and journals, hotspots, and frontiers in this field. England and Canada led the world in the field of CVD with frailty. NIA and McGill University were influential in CVD with frailty. Although Asian authors were prolific, the European and American authors still played a lead role; therefore, cooperation between countries/regions should be established. Hotspots in the CVD with frailty have been summarized, they were frailty in the elderly with CVD, exercise intervention, assessment for CVD patients with frailty, quality of life, and common diseases related to CVD with frailty. Frontiers in the CVD with frailty have been summarized as care and intervention of CVD patients with frailty, social frailty, and validation of CVD with frailty.

This study shows that there is a gap in the quality of research about CVD with frailty between developed countries/regions and developing countries/regions. It is needed to establish long-term and in-depth international medical cooperation. This will contribute to the sustainable development of this research domain in developing countries/regions, thereby putting more attention on CVD patients with frailty to improve outcomes. Screening the pre-frailty dwellers and taking some simple interventions are effective measures to reduce medical costs. Most importantly, world public health will benefit from establishing good cooperation.

There are hardly any researchers who will focus on the frailty of young people or children. In fact, some risk factors related to frailty may occur in young people or children, such as disability, obesity, inflammation, or some other chronic diseases. As the lifestyle and living environment change, CVD is gradually becoming more common among young people. However, in the existing studies, most studies focus on the elderly, but young CVD patients with frailty also should get attention. For example, at-risk adolescents and young adults (AYA) are a population that needs attention. The survivors of childhood severe diseases or patients who previously accepted radiotherapy or chemotherapy may probably be exposed to frailty. Compare to their siblings, childhood cancer survivors were more likely to have adverse health status (43). Krnavek et al. found that childhood cancer survivors were more likely to be pre-frail than people without cancer history (44). It is possible to have CVD and frailty in these special children when they grow up. Thus, it is necessary to explore the relationship between childhood adverse events and CVD with frailty, then decide whether to perceive childhood adverse events as a crucial risk factor for CVD with frailty. In future, an association between AYA with CVD and frailty may become a new research orientation.

CVD with frailty will be a hot topic for a long time, and more comprehensive research in this domain will help patients and medical staff to cope with it. Besides, frailty in the different CVD patient population is also an aspect worth exploring, such as women, disability, cognitive impairment, and so on.

## Data availability statement

The original contributions presented in the study are included in the article/supplementary material, further inquiries can be directed to the corresponding author.

## Author contributions

XB, LC, YWe, and YD made substantial contributions to the conception and design of the research. QS operated the CiteSpace software. YWa and YD searched the database and collected the records that meet the criteria. All authors read and approved the final manuscript.

## Conflict of interest

The authors declare that the research was conducted in the absence of any commercial or financial relationships that could be construed as a potential conflict of interest.

## Publisher's note

All claims expressed in this article are solely those of the authors and do not necessarily represent those of their affiliated organizations, or those of the publisher, the editors and the reviewers. Any product that may be evaluated in this article, or claim that may be made by its manufacturer, is not guaranteed or endorsed by the publisher.
